# Improving sample classification by harnessing the potential of ^1^H-NMR signal chemical shifts

**DOI:** 10.1038/s41598-018-30351-7

**Published:** 2018-08-08

**Authors:** Daniel Cañueto, Reza M. Salek, Xavier Correig, Nicolau Cañellas

**Affiliations:** 1 Metabolomics Platform, IISPV, DEEEA, Universitat Rovira i Virgili, Campus Sescelades, Carretera de Valls, s/n, 43007 Tarragona, Catalonia Spain; 2 European Bioinformatics Institute (EMBL-EBI), European Molecular Biology Laboratory, Wellcome Trust Genome Campus, Hinxton, Cambridge, CB10 1SD United Kingdom; 3 CIBERDEM, Spanish Biomedical Research Centre in Diabetes and Associated Metabolic Disorders, Madrid, Spain

## Abstract

NMR spectroscopy is a technology that is widely used in metabolomic studies. The information that these studies most commonly use from NMR spectra is the metabolite concentration. However, as well as concentration, pH and ionic strength information are also made available by the chemical shift of metabolite signals. This information is typically not used even though it can enhance sample discrimination, since many conditions show pH or ionic imbalance. Here, we demonstrate how chemical shift information can be used to improve the quality of the discrimination between case and control samples in three public datasets of different human matrices. In two of these datasets, chemical shift information helped to provide an AUROC value higher than 0.9 during sample classification. In the other dataset, the chemical shift also showed discriminant potential (AUROC 0.831). These results are consistent with the pH imbalance characteristic of the condition studied in the datasets. In addition, we show that this signal misalignment dependent on sample class can alter the results of fingerprinting approaches in the three datasets. Our results show that it is possible to use chemical shift information to enhance the diagnostic and predictive properties of NMR.

## Introduction

Metabolomics (or metabonomics) is the study of the metabolome in biofluids, cells or tissues extracted from animals and plants by characterizing the metabolic fingerprint or phenotype (or their underlying mechanisms) in a biological system^1,2^. ^1^H-NMR spectroscopy is a high-throughput technique that quantifies metabolite concentrations in a reliable and reproducible manner^3^. ^1^H-NMR data can be used to classify samples, so it is a powerful means for capturing diagnostic and predictive properties and has promising potential for personalized medicine^4^.

A metabolite can be characterized in an ^1^H-NMR spectrum by its characteristic pattern of signals. The metabolite concentration can be measured by estimating the area below any one of these signals. Likewise, each signal has a specific location determined by its chemical shift (the resonant frequency of its nucleus in a magnetic field). For example, lactate concentration can be quantified from a signal with a chemical shift located at 1.33 ppm or from another signal with a chemical shift located at 4.11 ppm^5^. The chemical shift (that is to say, the location in a spectrum) of signals is influenced by the pH and the ionic strength (mostly mediated by Ca^2+^ or Mg^2^ concentration)^+^ of the sample^6^. The information about pH and ionic strength given by the chemical shifts has already been proved to be beneficial for the quality control of fruit juice^7^. A recent article showed that the pH and ionic strength of human urine samples can be extrapolated from chemical shift information^8^. A wide range of diseases (e.g., tumours^9^) are characterized by metabolic alkalosis/acidosis^10^ or ionic imbalance^8^: these diseases could be better identified in the NMR data with the help of chemical shift information. In addition, theoretical proof of the potential of chemical shift information to separate samples is already available^11^. Even so, chemical shift information is still not used to characterize these sample properties and possible differences between classes because the pH and ionic strength can be masked by phosphate buffering and the dilution of matrices varies considerably. These factors hinder the interpretability of the pH information provided by DFTMP^12^ or Chenomx-based pH calibration.

To date, several tools have been developed to automatically quantify metabolite concentrations in 1D ^1^H-NMR spectra datasets^13–15^, making it easier to collect additional information, including signal chemical shifts. For example, a recent redesign of the Dolphin NMR tool rDolphin using open-source R language provided more flexible and reproducible automatic metabolite profiling in 1D ^1^H-NMR datasets^16^. One additional feature of rDolphin is its ability to capture and output additional information (such as the signal parameter values –including chemical shift– from every quantified signal) for further evaluation. The collection of multiple chemical shifts and the open-source availability of complex algorithms able to combine their information make it possible to use chemical shift information to discriminate samples despite the drawbacks of pH masking and dilution mentioned above. In this study, we report an approach to combine the binomial of metabolite concentration and signal chemical shift information in NMR data from metabolomic studies to maximize NMR discriminant potential. To do so, we quantified the metabolite concentrations and signal chemical shifts of three public NMR metabolomic study datasets. We found that chemical shift information can be used to separate samples more effectively than just metabolite concentration information.

## Materials and Methods

### Datasets

Three NMR datasets from different human matrices from MetaboLights^17^ (a public repository of metabolomic studies) were analysed and profiled:

- MTBLS1 Metabolights dataset: fingerprint NMR data (with adaptive binning) was used to analyse metabolomic changes mediated by type 2 diabetes in mouse, rat, and human urine^18^. The Metabolights dataset provides human urine data of 84 samples from nondiabetics and 48 samples from diabetics.
- MTBLS237 Metabolights dataset: in human faecal extract samples, fingerprint NMR data was used to determine the metabolic profiling of control subjects and patients with active or inactive ulcerative colitis (UC) and Crohn’s disease (CD)^19^. The spectra dataset analysed consisted of: 20 control samples, 14 active CD samples, 31 inactive CD samples, 19 active UC samples and 28 inactive UC samples.
- MTBLS374 Metabolights dataset: the metabolic serum profiles of smokers and nonsmokers were compared in order to study functional alterations caused by smoking through fingerprint data^20^. The original study analysed ^1^H-NMR fingerprint data, with the help of 2D spectrum information, to identify metabolites. According to the information available on the repository, the spectra dataset analysed in our study consisted of 56 samples from smokers and 57 samples from nonsmokers.

Details about sample preparation, spectrum acquisition and main results are available in the original manuscripts. Information about the buffer and dietary restrictions in the original studies is available in Supplementary Information. Information about chemical shift variability in metabolite signals after sample preparation is available in Supplementary Fig. 1. The ethical issues regarding the studies associated with the used datasets are described in detail in their original articles^18–20^.

### Spectra preprocessing and profiling

The spectrum preprocessing parameters available in the manuscripts of the studies associated with the datasets used were evaluated to generate ^1^H-NMR spectra similar to the ones of the original studies. All datasets were normalised using Probabilistic Quotient Normalisation (PQN) as it is the recommended normalisation method in recent reviews^21^. This method analyses the distribution of quotients of the amplitudes of each spectrum with those of a reference spectrum, and then normalises the spectrum by the median of the distribution of quotients^22^. Then, data binning (0.0006 ppm) was applied to the spectra before they were profiled by rDolphin. Unreliable relative metabolite concentrations and signal chemical shifts were filtered using a variety of quality indicators (additional information is available in Supplementary Information). Then, univariate outliers for each feature (controlling for sample class) were set as missing values and imputed.

For metabolite concentration information, the final dataset consisted of: MTBLS1, 39 features; MTBLS237, 35 features, MTBLS374, 30 features. For chemical shift information, the features were highly correlated. Consequently, in each dataset, dimensionality was reduced by principal components analysis (PCA) and the dozens of correlated chemical shifts were grouped into 5 independent principal components (enabling the factors influencing signal chemical shifts to be accurately evaluated).

### Multivariate analysis

First, an exploratory visualization was performed in both metabolite concentration and chemical shift information datasets to compare their discriminant potential. The visualization was based on the results of a PCA performed to each set of information. During this exploratory visualization, it was also checked that no batch effects exerted an effect on the observed differences.

Next, sample classification was performed using the random forest algorithm, a decision tree-based algorithm which combines predictions and uses bootstrapping to maximize the optimization of bias and variance^23,24^. The modelling workflow provided by the ‘caret’ R package was used to perform sample classification. The models were trained with an average number of 500 trees, automatic hyperparameter tuning to best adapt to data properties, 500-iteration 0.632 bootstrap resampling to avoid overfitting^25^, upsampling to maximize the robustness of the models against the class imbalance problem in datasets^26^, and recursive feature elimination to minimize the influence of non-informative features. Classification was performed in three different variable subsets: 1- Only relative metabolite concentrations, 2- Only signal chemical shifts and 3- Using both relative metabolite concentrations and signal chemical shifts. Results were evaluated using classification accuracy, Cohen’s kappa (a more robust indicator against chance classification and class imbalance) and the area under the receiver operating characteristic (AUROC). In addition, to further evaluate the trained models, the sensitivity, specificity, positive predicted value and negative predicted value are available in Supplementary Information. Lastly, the variable importance in the models generated with both sets of variables was measured.

### Reproducibility of study workflow

To validate and reproduce the results, the profiling output, the data analysis workflow and the links for downloading the datasets analysed are available on github.com/danielcanueto/chemical_shift_classification.

### Data availability

All the data and the study workflow are available on github.com/danielcanueto/chemical_shift_classification to ensure reproducibility.

## Results

### Exploratory visualization of PCA information

**Figure 1:**
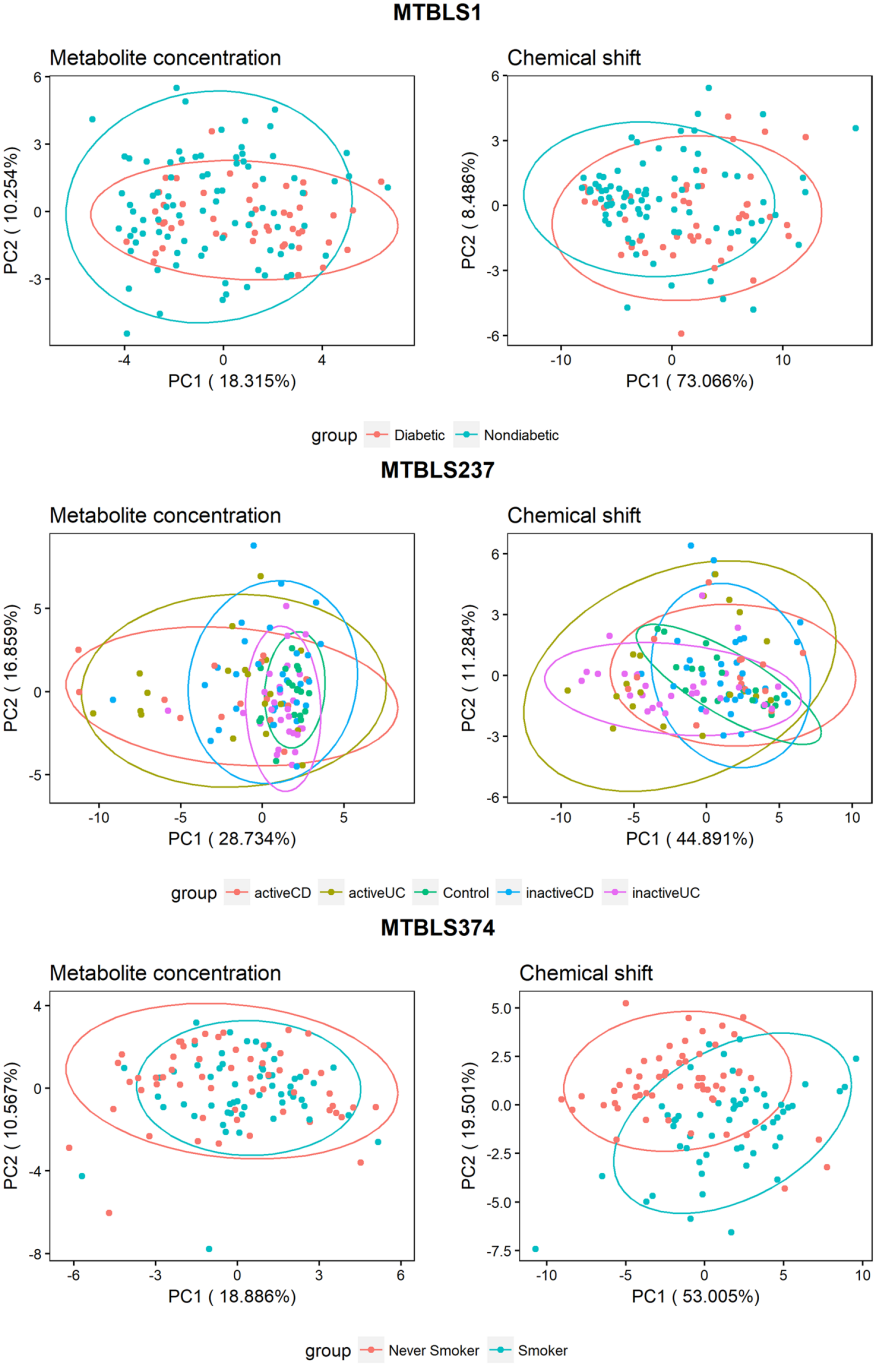
Exploratory PCA analysis shows the potential of the chemical shift data in the classificaton models. The first PCs of the PCA using chemical shifts (right) show better separation than the ones using concentrations (left). Plots also suggest no batch effects necessary to monitor.

Visualization of the first two principal components (PCs) of the PCAs of metabolite concentrations and signal chemical shifts suggested higher discriminant power in chemical shift information (Fig. 1). In chemical shift figures, less ellipse overlap (or at least more separated centres) was observed. Although more discriminative power in concentration information might be present in later PCs, the noise-related variance might be able to mask this power more intensely. Also, no batch effects were visible on any dataset.

### MTBLS1 dataset

Chemical shift information showed potential for discriminating between diabetic and non-diabetic samples during random forest classification (AUROC 0.831) (Table 1). However, adding chemical shift information did not improve the excellent results obtained with only metabolite concentrations (AUROC 0.979).

**Table 1:** Chemical shift information shows discriminative potential in the MTBLS1 dataset.

	Both sets of information	Concentration information	Chemical shift information
Accuracy	0.929	0.933	0.795
kappa	0.840	0.849	0.559
AUROC	0.980	0.979	0.831

However, it cannot enhance the excellent results given by concentration information during random forest classification.

### MTBLS237 dataset

Chemical shift information, alone or combined with metabolite concentration information, significantly improved sample discrimination in 6 of the 8 subgroup comparisons: Active UC vs Inactive UC (0.917 vs 0.811 in AUROC), Active UC vs Active CD (0.768 vs 0.743 in AUROC), Inactive UC vs Inactive CD (0.870 vs 0.810 in AUROC), Control vs Active UC (0.948 vs 0.914 in AUROC), Control vs Inactive UC (0.943 vs 0.823 in AUROC) and Control vs Inactive CD (0.854 vs 0.825 in AUROC) (Table 2).

**Table 2:** Adding chemical shift information to concentration information improved the classification between the five different kinds of sample in the MTBLS237 dataset.

	Both sets of information	Concentration information	Chemical shift information
	**Active UC vs Inactive UC**		
Accuracy	0.863	0.826	0.876
kappa	0.635	0.555	0.698
AUROC	0.870	0.811	0.917
	**Active CD vs Inactive CD**		
Accuracy	0.801	0.808	0.721
kappa	0.505	0.526	0.331
AUROC	0.768	0.777	0.661
	**Active UC vs Active CD**		
Accuracy	0.730	0.717	0.668
kappa	0.462	0.438	0.339
AUROC	0.768	0.743	0.682
	**Inactive UC vs Inactive CD**		
Accuracy	0.808	0.771	0.797
kappa	0.617	0.545	0.594
AUROC	0.870	0.810	0.841
	**Control vs Active UC**		
Accuracy	0.890	0.860	0.882
kappa	0.773	0.714	0.762
AUROC	0.948	0.914	0.926
	**Control vs Active CD**		
Accuracy	0.867	0.861	0.790
kappa	0.719	0.707	0.556
AUROC	0.921	0.916	0.839
	**Control vs Inactive UC**		
Accuracy	0.882	0.804	0.892
kappa	0.753	0.596	0.775
AUROC	0.926	0.823	0.943
	**Control vs Inactive CD**		
Accuracy	0.806	0.787	0.782
kappa	0.589	0.550	0.551
AUROC	0.854	0.825	0.81

Several quality indicators of the models generated with only concentration information, only chemical shift information and both sources of information are shown for the eight comparisons between the five subclasses (control, active UC, inactive UC, active CD, inactive CD).

### MTBLS374 dataset

Random forest classification on smoker and nonsmoker samples showed much higher AUROC values with chemical shift information than with metabolite concentration (0.937 vs 0.856 in AUROC) (Table 3). The combination of both sources of information gave slightly better values than when only chemical shift information was used (AUROC 0.950; Table 3, left).

**Table 3:** Adding chemical shift information to concentration information provides the best classification of samples in the MTBLS374 dataset.

	Both sets of information	Concentration information	Chemical shift information
Accuracy	0.899	0.806	0.883
kappa	0.797	0.614	0.766
AUROC	0.950	0.856	0.937

Several quality indicators of the models generated only with concentration information, only with chemical shift information and with both sources of information are shown.

## Discussion

The results of our studies showed that 1D ^1^H-NMR spectra chemical shift information can give greater insight into sample properties and improve sample classification. In the three datasets analysed, chemical shift information led to good sample classification. In addition, in two of them, chemical shift information helped gave AUROC values higher than 0.9 and improved the classification with only metabolite concentration information.

### Relationship between chemical shift and metabolic alkalosis/acidosis

The high classification performance observed in the three study datasets seems to be consistent with what has been previously reported about the alkalosis or acidosis characteristics of the conditions in the associated studies.

The MTBLS1 dataset is associated with the study of the changes in human urine caused by type 2 diabetes. Type 2 diabetes mediates lower pH in urine as a result of greater net acid excretion and fewer ammonia buffers^27^. A lower pH increases the chemical shift of signals (i.e., the signal moves to the left in a spectrum)^28^. Accordingly, most signals show a higher chemical shift in the diabetes samples than in the control samples (Supplementary Fig. 2, top). Several signal chemical shifts (such as one of indoxyl sulfate in Supplementary Fig. 2) show an inverse trend to the other signals. This inverse trend may be mediated by the influence of ionic strength. However, it may also be an artefact of the TSP signal used to reference spectra. The pKa of TSP is approximately 5, which makes its signal chemical shift sensitive to pH variation and causes signals with lower sensitivity (like the ones in the phenolic region^29^) to seem to move in the opposite direction to other signals.

**Figure 2:**
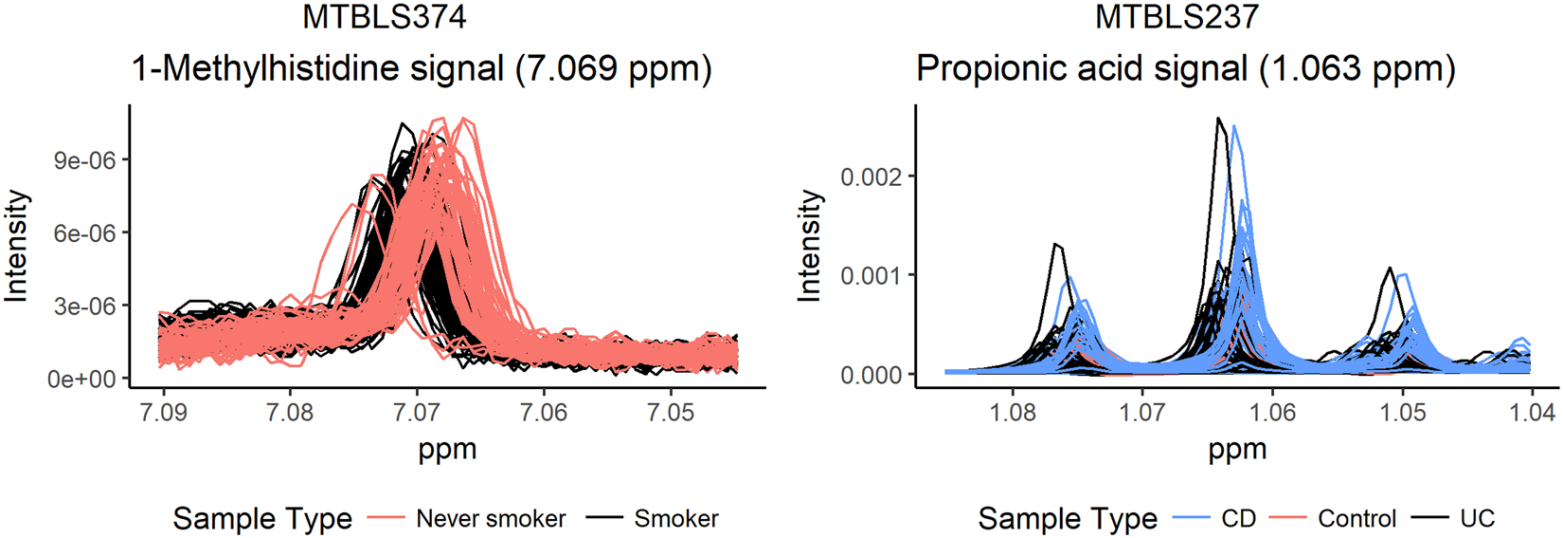
Signals can be misaligned in some sample classes. Low pH mediated by the condition studied increases the chemical shift of the signals. The resulting class-dependent signal misalignment can distort the results of the analysis of fingerprint data: features can show significant differences caused by differences in chemical shift (mediated by pH or ionic strength) rather than by differences in metabolite concentration.

In the case of the MTBLS237 dataset, alkalosis/acidosis in inflammatory bowel disease (the subtypes of which are UC and CD) has been reported elsewhere in the literature^30^. The relationship between faecal pH and the disease could be influenced by the location of lesions and/or the complex acid-base balances. The pH disturbance could have manifested as acidic pH in the UC samples represented by a higher chemical shift (Fig. 2, right; Supplementary Fig. 2, middle), and has been reported in the literature^31^. As in the MTBLS1 dataset, several signal chemical shifts show an inverse trend that may be mediated by the use of the TSP signal to reference spectra (Supplementary Fig. 2, middle).

As for the MTBLS374 dataset, respiratory acidosis is typically seen in lung disease developed by smokers^32^ and in cigarette smoke that contains oxidants with acidic properties^33^. Signals in the spectra from the smokers group showed a higher chemical shift than the equivalent signals in the non-smokers (Fig. 2, left; Supplementary Fig. 2, bottom). This effect might be mediated by a more acidic pH in smokers’ samples as a consequence of smoking, which would be mostly captured by the second principal component of the PCA of signal chemical shifts (Supplementary Table 1). Unlike the other two datasets, this dataset does not contain any signal chemical shift with an inverse trend. This is consistent with the reference signal being glucose, a metabolite with a pKa (approx. 12) that is quite different from the pH of biological samples and thus much more resilient to pH variability.

### Effect of class-dependent signal misalignment on fingerprinting approaches

All the datasets evaluated were processed using fingerprinting approaches in the original studies, in contrast to the profiling approach used here. Fingerprinting approaches perform the classification by looking for significant spectral differences between groups and identifying the metabolites involved in the second stage. On the other hand, profiling approaches start by characterizing the metabolites in the samples and then performing statistical analysis in the second stage. Their different workflows imply variations in how metabolites are identified and how their concentrations are quantified^34^.

Profiling is deemed to provide more resistance against signal overlap or baseline appearance through the deconvolution of signals in the spectrum lineshape^35^. However, one factor not evaluated in the differences between fingerprinting and profiling approaches is class-dependent signal misalignment (i.e., the differences in signal chemical shifts between spectra from different sample classes). Fingerprinting reliability is based on the premise that signals are reasonably well-aligned throughout the spectra dataset and, consequently, the differences are caused by differences in metabolite concentrations. It has been theoretically demonstrated that classification in fingerprint data can be influenced by class-dependent signal misalignment (i.e, that the differences found between classes are actually caused by having the metabolite signals located in different bins). However, approaches to minimize this problem (like the use of signal alignment algorithms^36^) are still not prevalent in the metabolomics field and were not applied in any of the datasets analysed.

In the three datasets analysed, the results of the univariate analysis in fingerprint data were compared before and after signal alignment using the CluPA algorithm^37^ (the analysis workflow is available in Supplementary Information). Signal alignment decreased the number of significant bins in all datasets (MTBLS374, −42%; MTBLS1, −7%; MTBLS237, −5%). This decrease means an improvement in the quality of classification models, as it can be ensured that the differences between classes are caused by potential biomarkers and not by signal misalignment.

Results confirmed the effect that class-dependent signal misalignment can exert on the results of fingerprinting data. Therefore, they further recommend the adoption of profiling approaches enabled by recent open-source profiling tools to minimize the generation of non-reproducible results. If the fingerprinting approach is still preferred, the implementation of signal alignment algorithms can minimise non-reproducible results; nonetheless, this alignment will involve losing the information given by chemical shift information.

### Future directions and challenges

Our study workflow uses publicly available datasets and performs data preprocessing, profiling and statistical analysis with open-source tools following community recommendations^38^. By sharing this workflow we hope to make the use of chemical shift information in NMR studies more straightforward and more widespread. In addition, we hope the resulting reproducibility helps assess some aspects that need to be taken into account to take maximum advantage of chemical shift information:

- Some matrices present considerable variations in dilution, which can greatly influence their pH and ionic strength (and, therefore, chemical shift). In addition, chemical shift variability is reduced by adding phosphate buffers (sometimes with added chelators such as EDTA) to the sample^39^. Both dilution variability and the use of buffers may mask the effects on the chemical shift produced by the condition studied. Consequently, the fact that the discriminative potential observed in MTBLS1 and MTBLS237 datasets was lower than the potential of the MTBLS374 dataset may be due to the higher dilution variability in the matrices studied (human urine and faecal extracts). The use of buffers or chelators should be minimized and sample dilution variability should be reduced if maximum advantage is to be taken of the properties of chemical shift information.
- It has been suggested that chemical shift information could also be translated to sample pHs and ionic concentrations, hence maximizing the information extracted from a dataset^8^. Nonetheless, the limitations mentioned above raise concerns about the correct use of this information in several commonly studied matrices. In addition, the fact that these matrices commonly use a signal to reference spectra that is not resilient to pH (such as the TSP signal) may further distort the translation of chemical shifts to pH and ionic concentration values. There are several affordable techniques (e.g., pH meter or potentiometer) for directly measuring pH and ion concentrations that make this challenging translation unnecessary.
- Studies aiming to take advantage of chemical shift information should ensure consistent sample preparation and spectra acquisition in all samples in order to prevent the discrimination between sample classes being mediated by differences in the preparation or acquisition protocol.
- Further improvements in the quality of the classification models generated may be made by extracting more chemical shifts from NMR datasets and filtering noise in the chemical shift information (caused by low resolution with the consequent signal overlap in ^1^H-NMR) prior to model training. High-resolution spectra (e.g., 2D NMR) could help isolate more signals (with their associated chemical shifts) from different nuclei and prevent noise.

## Electronic supplementary material

Supplementary Information
