# Supplementary material for: Improving sample classification by harnessing the potential of 1H-NMR signal chemical shifts
Source: Sci Rep. 2018 Aug 8;8:11886. doi: 10.1038/s41598-018-30351-7 (PMC6082897; doi:10.1038/s41598-018-30351-7)
Supplement: Supplementary file 1 — Supplementary Information [file 41598_2018_30351_MOESM1_ESM.docx]

**Supplementary Information of:**

Improving sample classification by harnessing the potential of ^1^H-NMR signal chemical shifts

## Daniel Cañueto*^1^, Reza M. Salek^2^, Xavier Correig^1,3^, and Nicolau Cañellas^1,3^

**Information about buffer and dietary restrictions in the original studies.**

Buffer:

- MTBLS1: 400 μl human urine sample aliquots +200 μl phosphate buffer (0.2 M, pH 7.4). Then, 500 μl supernatant after centrifugation + 100 μl of sodium TSP/D2O/sodium azide solution (0.05% wt/vol TSP in D2O and 1% wt/vol sodium azide).
- MTBLS237: Faecal stool homogenized adding 2 volumes (w/v) of sterile phosphate buffered saline (PBS 1.9 mM Na2HPO4, 8.1 mM NaH2PO4,150 mM NaCl, pH 7.4). Then, 40 μl of faecal extract + 4 μl of D2O/500 μM TSP.
- MTBLS374: 300 μl serum sample + 300 μl phosphate buffer (62.5 mM NaH2PO4, pH 7.4).

Dietary restrictions:

- MTBLS1: control patients abstained from using prescription and non prescription drugs for 14 days (or five half lives, whichever was longer). Diabetics stopped taking medication during the study, and samples were collected after a four-week drug-free wash-out period. They followed the American Diabetes Association dietary guidelines and abstained from caffeine- and xanthene-based products for 24 hours prior to and during the study. All volunteers abstained from alcohol and had their diet controlled throughout the study.
- MTBLS237: exclusion within 14 days of probiotics, and special food diets (e.g, FODMAP, diabetic diets, or gluten-free diet).
- MTBLS374: exclusion of caffeine, alcohol, and grilled, fried and smoked food 48 h before sample collection.

**Filtering of unreliable metabolite relative concentrations and chemical shifts.**

- First, relative concentrations and chemical shifts of signal quantifications which did not pass a specific threshold in two quality indicators outputted by rDolphin (fitting error, signal area / total spectrum area ratio) were removed.
- Then, outliers for each signal area quantification (controlling by sample type) were removed.
- Next, relative concentrations and chemical shifts with 40% missing values or more were removed from the analysis.
- When more than one signal was quantified for a metabolite, the signal with the lowest number of missing values was considered to be the one able to provide the most accurate relative concentration and the quantification of the other signals was removed.
- Finally, missing values from chemical shifts and relative concentrations were imputed by random forest methods.

**Filtering of non-informative signal chemical shifts.** Inaccurate chemical shift quantification in multiplet integration or lack of meaningful chemical shift variability mediated the presence of chemical shifts with noisy (i.e., non-informative) information in the dataset. Chemical shifts are correlated so an internal consistency in the chemical shift dataset is expected and this consistency can be measured. This internal consistency was analysed with the ‘psych’ R package. The chemical shifts which worsened the internal consistency of the chemical shift dataset were removed.

**Univariate tests in non-aligned and aligned fingerprint data.** The ‘p_values’ function of the [‘rDolphin’](https://github.com/danielcanueto/rDolphin) R package contains the workflow that generates univariate tests for every bin. In two-sample tests, non-normality in every group of samples is checked using Shapiro-Wilk tests. If a group of samples shows no normality, a Mann-Whitney test is performed; if all groups show normality a Welch t-test is performed (to understand why Welch and not Student’s t-tests are performed, see this [link](http://daniellakens.blogspot.com.es/2015/01/always-use-welchs-t-test-instead-of.html)). The p-values estimated in every study were then Benjamini-Hochberg adjusted.

**Supplementary Figures**


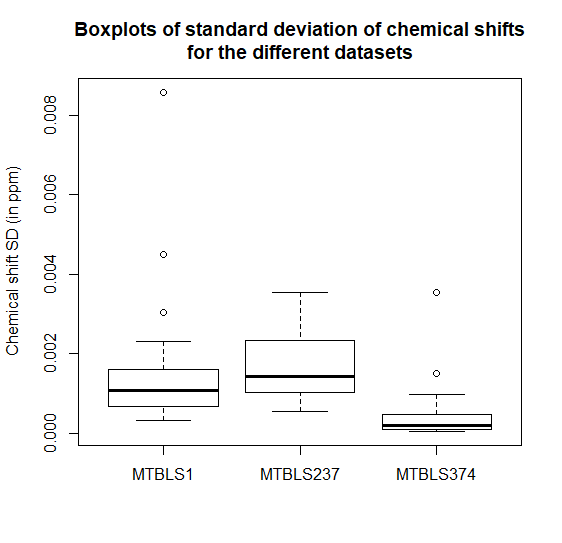


**Supplementary Figure 1.** Variability (measured by standard deviation) of the chemical shifts analysed in the three datasets. As expected, the dataset of human matrices with higher dilution variability (urine and faecal extracts) show higher chemical shift variability. In all three datasets, the use of buffers does not impede the appearance of chemical shift variability that can be analysed.


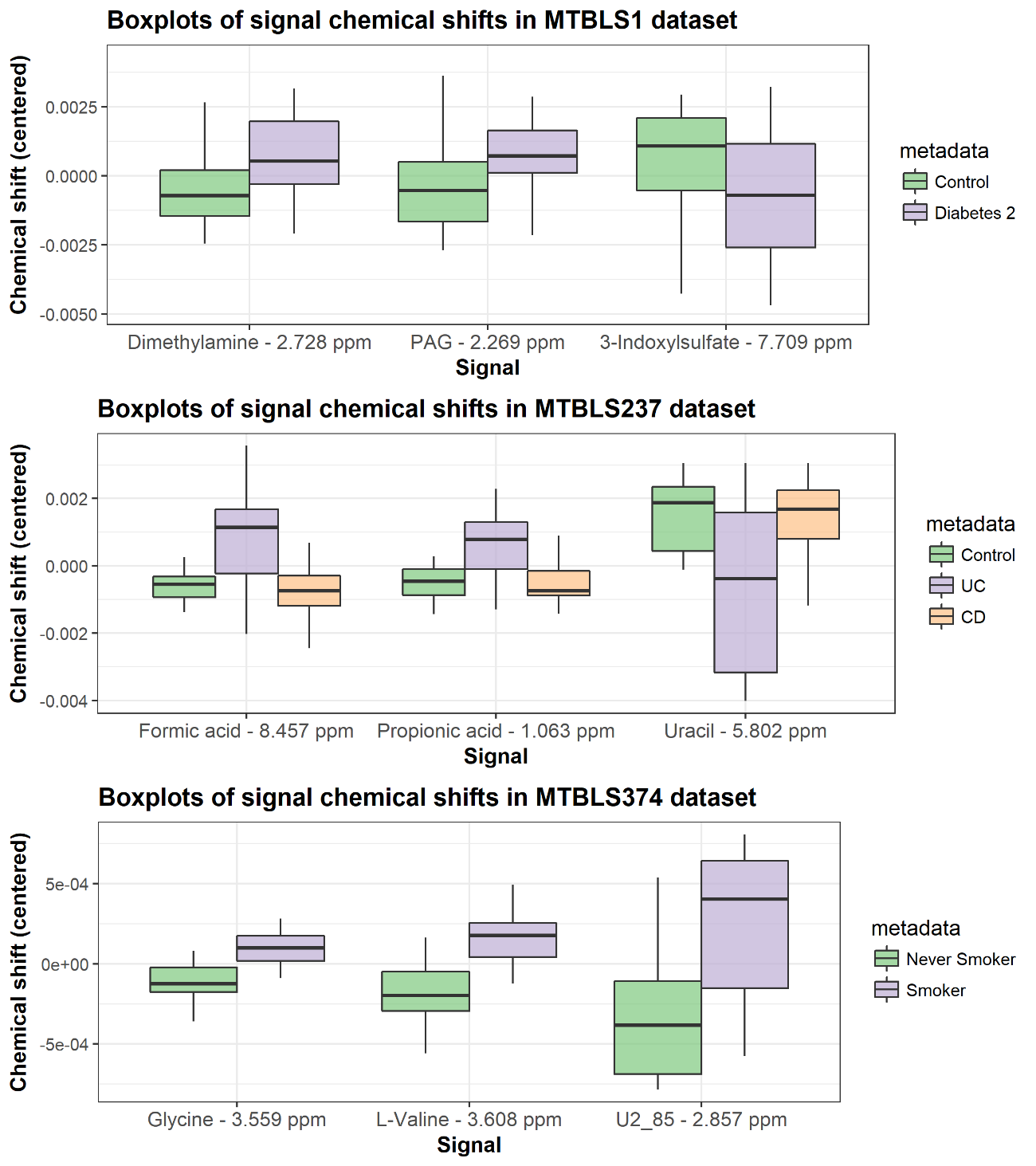


**Supplementary Figure 2. Distribution of centred chemical shift of three good chemical shift predictors in the MTBLS1 (top),** **MTBLS237 (middle) and MTBLS374 (bottom) datasets.** Chemical shift patterns in the MTBLS1 and the MTBLS237 datasets showed higher complexity (with some signals with inverse trends) in the chemical shift mediated by the use of TSP as reference.

**Supplementary Tables**

| **Predictors** | **Importance** | |  |  |
| --- | --- | --- | --- | --- |
| PC2 - Chemical shift | | | 100 | |
| PC1 - Chemical shift | | | 56.4 | |
| Citric.acid - quantification | | | 32.181 | |
| U2_85 - quantification | | | 24.541 | |
| PC3 - Chemical shift | | | 24.39 | |

**Supplementary Table 1.** **Ranked predictors in random forest classification of samples with both concentration and chemical shift information in the MTBLS374 dataset**. There are few predictors because of the recursive feature extraction of non-discriminative features.

|  | **Both sets of information** | **Concentration information** | **Chemical shift information** |
| --- | --- | --- | --- |
| **Sensitivity** | 0.838 | 0.855 | 0.713 |
| **Specificity** | 0.986 | 0.982 | 0.841 |
| **Pos Pred Value** | 0.968 | 0.963 | 0.723 |
| **Neg Pred Value** | 0.915 | 0.923 | 0.835 |

**Supplementary Table 2. Additional classification indicators in the MTBLS1 dataset.**

|  | **Both sets of information** | **Concentration information** | **Chemical shift information** |
| --- | --- | --- | --- |
|  | **Active UC vs Inactive UC** | | |
| **Sensitivity** | 0.76 | 0.751 | 0.6 |
| **Specificity** | 0.842 | 0.882 | 0.806 |
| **Pos Pred Value** | 0.776 | 0.815 | 0.658 |
| **Neg Pred Value** | 0.837 | 0.842 | 0.755 |
|  | **Active CD vs Inactive CD** | | |
| **Sensitivity** | 0.595 | 0.616 | 0.483 |
| **Specificity** | 0.909 | 0.909 | 0.839 |
| **Pos Pred Value** | 0.725 | 0.745 | 0.516 |
| **Neg Pred Value** | 0.838 | 0.845 | 0.789 |
|  | **Active UC vs Active CD** | | |
| **Sensitivity** | 0.674 | 0.662 | 0.617 |
| **Specificity** | 0.798 | 0.783 | 0.721 |
| **Pos Pred Value** | 0.702 | 0.684 | 0.615 |
| **Neg Pred Value** | 0.781 | 0.769 | 0.725 |
|  | **Inactive UC vs Inactive CD** | | |
| **Sensitivity** | 0.871 | 0.837 | 0.852 |
| **Specificity** | 0.847 | 0.816 | 0.851 |
| **Pos Pred Value** | 0.864 | 0.835 | 0.865 |
| **Neg Pred Value** | 0.861 | 0.824 | 0.846 |

|  | **Both sets of information** | **Concentration information** | **Chemical shift information** |
| --- | --- | --- | --- |
|  | **Active UC vs Inactive UC** | | |
| **Sensitivity** | 0.866 | 0.839 | 0.882 |
| **Specificity** | 0.915 | 0.885 | 0.891 |
| **Pos Pred Value** | 0.912 | 0.882 | 0.895 |
| **Neg Pred Value** | 0.881 | 0.858 | 0.888 |
|  | **Active CD vs Inactive CD** | | |
| **Sensitivity** | 0.828 | 0.821 | 0.671 |
| **Specificity** | 0.901 | 0.897 | 0.885 |
| **Pos Pred Value** | 0.86 | 0.854 | 0.794 |
| **Neg Pred Value** | 0.883 | 0.878 | 0.802 |
|  | **Active UC vs Active CD** | | |
| **Sensitivity** | 0.842 | 0.739 | 0.889 |
| **Specificity** | 0.917 | 0.863 | 0.897 |
| **Pos Pred Value** | 0.876 | 0.793 | 0.868 |
| **Neg Pred Value** | 0.894 | 0.829 | 0.921 |
|  | **Inactive UC vs Inactive CD** | | |
| **Sensitivity** | 0.744 | 0.708 | 0.741 |
| **Specificity** | 0.852 | 0.848 | 0.82 |
| **Pos Pred Value** | 0.768 | 0.751 | 0.735 |
| **Neg Pred Value** | 0.841 | 0.824 | 0.832 |

**Supplementary Table 3. Additional classification indicators in the MTBLS237 dataset.**

|  | **Both sets of information** | **Concentration information** | **Chemical shift information** |
| --- | --- | --- | --- |
| **Sensitivity** | 0.893 | 0.810 | 0.870 |
| **Specificity** | 0.907 | 0.805 | 0.887 |
| **Pos Pred Value** | 0.906 | 0.809 | 0.886 |
| **Neg Pred Value** | 0.895 | 0.810 | 0.874 |

**Supplementary Table 4. Additional classification indicators in the MTBLS374 dataset.**
